# Challenges of implementation of hospital accreditation in Iran: an exploratory factor analysis

**DOI:** 10.1186/s42506-019-0033-6

**Published:** 2020-02-18

**Authors:** Narges Tashayoei, Pouran Raeissi, Amir Ashkan Nasiripour

**Affiliations:** 1 Department of Health Services Management, School of Medical Sciences, Tehran Science and Research Branch, Islamic Azad University, Tehran, Iran; 2 Department of Health Services Management, School of Health Management and Information Sciences, Iran University of Medical Sciences, Tehran, Iran

**Keywords:** Accreditation, Challenges, Hospital, Iran

## Abstract

**Background:**

Today, accreditation is considered as the most commonly used health assessment approach. Considering the importance and application of the process of this approach in the hospital, this study aimed to investigate the challenges of implementation of hospital accreditation in Iran using exploratory factor analysis.

**Methods:**

A cross-sectional study design was carried out from July to December 2017, consisting of 200 managers in the seven hospitals accredited by the MOH in Tehran, Iran. Samples were selected through the purposive sampling method, and data were collected using a structured questionnaire in which validity and reliability were confirmed. Likert’s five-choice range was used for the rating of items. Exploratory factor analysis was used to categorize the identified challenges and extract the mathematical model on them.

**Results:**

Exploratory factor analysis identified six dimensions (implementation, evaluation, content, structural, psychological, and managerial) with 40 items using a 5-point Likert scale. Each dimension accounted for greater than 63.20% of the total variance. The scale had strong content validity (indices = 0.84). Each dimension of Cronbach’s alpha ranged from 0.74 to 0.94. Implementation, evaluation, content, structural, psychological, and managerial components also formed the final identified areas.

**Conclusion:**

The present study showed that there were major challenges in the path to successful deployment of Iranian hospital accreditation, requiring serious action by managers and policymakers in this field.

## Introduction

In today’s world, quality of health services is one of the main issues on the agenda of governments and health systems of different countries, especially in the countries of the Eastern Mediterranean [1]. Several factors require the implementation of quality promotion programs in the health system, including the increasing costs of clinical technologies, inadequate safety of care provided, and changing patterns of health and demography in the world [1–3]. Accreditation is one of the main approaches to improving quality in health systems worldwide [4] which is expanding at a fast pace [5]. Currently, accreditation programs are in use in all areas of the health system, such as acute care, primary care, and the elderly [6, 7].

Accreditation is the process of self-assessment and external evaluation by professional counterparts on the basis of optimal standards, with the aim of accurately assessing and continuously improving the performance of health organizations [1]. This approach seeks to provide quality and safe healthcare services and subsequently improve health outcomes [8]. Several researches introduce accreditation as the first step in achieving excellence in health care [9], and acquiring certification is a symbol of the quality of organizational performance [10]. In this regard, the capacity of accreditation programs to make positive changes and promote evidence-based clinical and organizational activities has led to its acceptance as a driving force in advancing and improving the health system [11].

Accreditation has features that make it preferable to lawmakers, service providers, insurers, and payers as well as recipients of services for other evaluated approaches. One of these excellences can be the comprehensiveness and multiplicity of the evaluation, the conformity of assessment methods with the specific nature of the health system, attention to promotion as the main objective of evaluation, and the use of expert evaluators [12]. The studies show the positive impact of accreditation in enhancing effectiveness, efficiency, quality, safety, leadership, teamwork, and communication, as well as satisfaction and service providers [1, 13, 14]. Also, accreditation could provide an opportunity for increasing funding [15] or reducing costs [16].

However, the use of accreditation in many areas has led to controversial views on users, because some of them powerfully support the values and benefits of it and others are concerned about its costs and the relevance of standards to clinicians and instability in evaluators [17–19]. The results of a study in Denmark suggest a negative attitude of hospital personnel towards accreditation, as this approach led to the creation of maladministration, excessive documentation, excessive pressure on employees, and the unnecessary focus on unnecessary processes [17]. In another study, it was claimed that the difference between the admission rate and the re-admission rate in successful and unsuccessful hospitals was not found in the confirmation of accreditation [20].

Iranian accreditation is a mandatory accreditation system. This system was established in 2010 by the Ministry of Health and was implemented in two phases. The first standards were tested in 2011. The second phase launched in 2012 and all public and private hospitals have been accredited [1].

Therefore, the mere implementation of accreditation programs cannot lead to positive changes because many factors can lead to a lack of accreditation to achieve predetermined goals and the potential benefits of it. Some of the reasons for these contradictions can be found in the underlying factors such as the type of occupation and profession, the accreditation model used, and the organizational, financial, and political barriers [1, 17–19]. It is clear that environmental factors such as organizational culture, education level, motivation, and existing rules and regulations can have a significant impact on the success rate of accreditation programs [1, 17]. To further understand and identify challenges of hospital accreditation, we conducted an exploratory factor analysis of the baseline questionnaire responses. Factor analysis is a statistical technique that reduces a large number of interrelated questions to a smaller number of underlying common factors or domains that are primarily responsible for covariation in the data [21]. Identifying the challenges faced by accreditation programs and providing upgrade solutions can have a huge impact on the success of these programs. Moreover, the Iranian hospital accreditation is in its infancy. Therefore, this study was designed to identify the challenges of its establishment in hospitals of Tehran, Iran.

## Methods

### Study design

We executed a cross-sectional survey.

### Setting

This study was conducted between July and December 2017 in seven hospitals in Tehran, Iran. Seven hospitals with at least 100 beds which are accredited by the MOH in Iran were purposively selected. These hospitals include three public hospitals, two social security, and two private hospitals.

### Study population

The participants consisted of the managers of hospitals at two levels of senior managers (chief executive officer, chief operating officer, and nursing manager) and middle managers (supervisors, quality improvement and accreditation managers, and heads of division).

### Study sample

The sample size in the study was determined using the common statistical method (sample size = five items the number of questionnaire questions) [22]. In this regard, based on the existence of 40 questions in the questionnaire, the sample size was calculated to be 200 people.

### Study tool

To identify and generate items appropriate for challenges in the instrument, we conducted a review of literature [1, 23–25]. A total of 112 items were extracted from the literature. We reduced the list down to 75 items after merging similar items and removing the duplicates.

Next, we invited experts and asked them to select most important for challenges for implementing accreditation in Iran. The experts reviewed the final list and ranked them in terms of importance and using a 1–9 point Likert scale, in which score one denoted as the least important and score 9 denoted as the most important challenges for implementing accreditation in Iran. The final list was contained 58 items.

A value of item-level content validity index (I-CVI) was assessed by ten experts, who were asked [1] to give suggestions on the relevancy of each item to the definition [2], to evaluate clarity and conciseness of the wordings. These experts were purposively recruited from the area of interest of this study. Among them were four experts in quality improvement and accreditation, two lecturers with expertise in accreditation, and four hospital managers.

The evaluation followed the process suggested by Polit et al. [26], in having experts rate each item on a 4-point Likert scale (not relevant, somewhat relevant, quite relevant, and very relevant) based on item clarity and conciseness.

The ratings were used to calculate an I-CVI and to determine if items should be revised or deleted. A criterion of 0.80 of I-CVI among the experts was selected for inclusion in the list of items [26]. During this step, content validity of the items with a score of 0.84% was approved for the I-CVI indices. Finally, 18 items were deleted regarding an acceptable value and 40 items were retained.

A pilot test of the preliminary instrument with a 5-point Likert scale (1 = very low and 5 = very high) was conducted on hospital managers that have similar characteristics of the sample.

Fifty-one samples were selected to complete the pilot survey. Cronbach’s alpha coefficient was examined to determine the internal consistency of the scale which indicates how well the items fit together conceptually [27], with the acceptable value of ≥ 0.70 [28]. The Cronbach’s alpha coefficient ranged from 0.74 to 0.94.

### Data analysis

Data was coded, tabulated, and analyzed using the SPSS software (SPSS for Windows, Version 20.0. Chicago, SPSS Inc.). Frequency (percentage) and the mean (standard deviation) were used to describe the data. Exploratory factor analysis was used to identify the existing structure and to determine the number and nature of challenges describing the covariance structure of these data. Statistical significance was based on *P* < 0.05.

## Results

### Sample demographics

The characteristics of the study sample are shown in Table 1. The majority of participants in this study were in the age range of 41–50 years (54.5%), males (76.5%), and graduated with bachelor’s degree (62.5%). The vast majority of managers were in the middle level (91.5%), and 66.5% of the sample had work experience of less than 20 years. The highest proportion of the respondents was working in public hospitals (40.5%).

**Table 1:** Demographic characteristics of managers, Iran hospitals, 2017

Characteristics	*N* (%)
Gender	
Male	153 (76.5)
Female	47 (23.5)
Age (year)	
30–40	69 (34.5)
41–50	109 (54.5)
51–60	21 (11.0)
Educational level	
Bachelor	125 (62.5)
Masters or PhD degree	75 (37.5)
Management level	
Senior (CEO, COO, nurse manager)	17 (8.5)
Middle (heads of division, quality improvement managers)	183 (91.5)
Work experience	
≥ 20	133 (66.5)
< 20	67 (33.5)
Hospital ownership	
Public	81 (40.5)
Social security	75 (37.5)
Private	44 (22.0)

In this study, a total of 40 challenges were identified for the implementation of hospital accreditation, which were entered into the final questionnaire after confirmation of validity and reliability. The results of the review of the participants’ viewpoints on the extent of the existence of the challenge are presented in Appendix. It should be noted that the score obtained by the variables is between 0 and 5; the score of 0 represents the absence of the problem in relation to the variable, and score 5 indicates a very significant challenge in relation to the relevant variable.

Accordingly, the variables “lack of time to implement the standards “, “a large number of measures which create confusion in the staff”, and “overemphasis on documenting and obtaining concessions only through documentation” were identified as the most significant challenges, with scores of 4.39, 4.37, and 4.35, respectively. Also, variables such as “the inappropriateness of the approach of evaluators to participants”, “compulsion to deceive the evaluators”, and “the interest of the evaluator and non-outsourcing” were rated as the weakest of the challenges with 2.32, 2.40, and 2.75 points, respectively. The average overall condition of the checklist variables was 3.64.

In order to categorize and extract the mathematical model governing the identified challenges, the exploratory factor analysis test was used. In this regard, at first, the data form and their appropriateness were analyzed for this analysis and two important criteria were used to examine the feasibility of factor analysis, namely, the adequacy index of the data from the Kaiser-Meyer-Olkin test (KMO) and Bartlett’s test. The KMO test is done to examine the adequacy of the sample size for factor analysis. In this study, the adequacy of the sample size with the score of 0.849 from this test (and higher than 0.70) is confirmed. Considering the fact that the feasibility of factor analysis also depends on the significance of the Bartlett test, this test was also performed on the data. The result of the Bartlett test (5060.924), which is less than 0.01 significant at the error level showed that the correlation matrix between the terms was not an equivalence matrix, and therefore, there is the ability to perform factor analysis. In addition, the KMO test was used to identify the total contribution of factors in the form of a common share table, and the results of this study showed that all research items (i.e., the identified challenges) had a common variance of more than 60% with other items and could explain the relevant variables.

Then, ten variables were extracted using Varimax rotation with a specific value higher than one. Therefore, out of the total of 40 variables (i.e., challenges), ten factors or extractions were extracted. Table 3 shows the value of the variance explained by the extracted factors after Varimax rotation. The contribution of each factor (descending order) varies in explaining the variance; thus, the first factor had the highest contribution (31.54% with special value 12.61) and factor 10, the lowest contribution (2.59% with special value of 1.03) in explaining the variables of the study. Also, the analysis shows that there is a direct relationship between the specific value and the explanatory share of each factor, which means that the factors that have a higher specific value have a larger share in explaining changes in variables (items). In sum, all ten factors with special values higher than 1 have been able to explain 70.52% of the variance of 40 items (questions) related to the scale of the accreditation challenges (Table 2).

**Table 2:** Percentage of variance explained by extracted factors after Varimax rotation

Factors	Initial Eigenvalues			Rotation Sums of Suqured Loadings		
	Total	Percentage of variance	Cumulative percentage	Total	Percentage of variance	Cumulative percentage
1	12.62	31.54	31.54	12.62	31.54	31.54
2	3.18	7.95	39.49	3.18	7.95	39.49
3	2.65	6.62	49.12	2.65	6.62	49.12
4	1.84	4.59	50.71	1.84	4.59	50.71
5	1.72	4.29	54.99	1.72	4.29	54.99
6	1.48	3.71	58.70	1.48	3.71	58.70
7	1.31	3.26	61.97	1.31	3.26	61.97
8	1.23	3.07	65.04	1.23	3.07	65.04
9	1.17	2.92	67.97	1.17	2.92	67.97
10	1.031	2.59	70.56	1.031	2.59	70.56

**Fig. 1:**
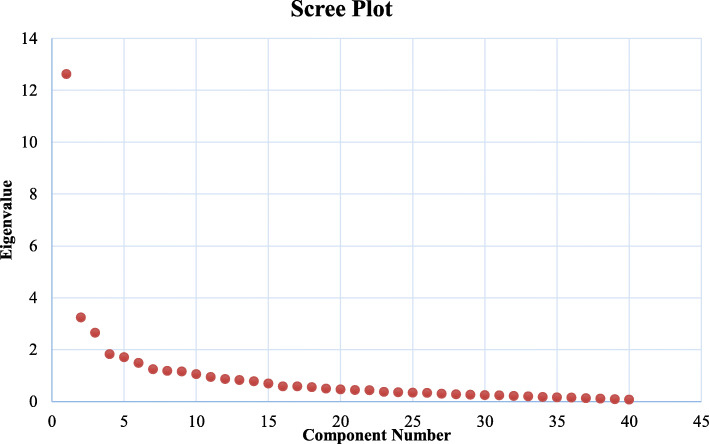
Factor analysis of Scree Plot

Also, the results of the KMO Factor Verification test are presented in Fig. 1 (known as “Crushed Stone”), which illustrates the number of suitable agents. The diagram shows that ten factors with a specific value are higher than 1, meaning that 40 questions and challenges related to each other are aggregated in ten factors.

Subsequently, alternative models were studied, and the number of factors was reduced one by one in order to select the appropriate model, and finally, a six-factor model based on the early theory of research, the Scree Plot result, and the data that could be interpreted as a prime model was identified, which explained 58.190 variance of the data. As a number of variables have been loaded onto more than one factor, therefore, at this stage, for ease of interpretation of data, variables that had a moderate factor-loading on different factors, that is, on more than one factor, were eliminated one by one. During the three phases of factor analysis, nine variables including variables 7, 8, 18, 20, 25, 28, 29, 35, and 36 from the sum of variables were eliminated, and the factor analysis test was performed again with 31 remaining variables. Finally, a six-factor model with 31 variables was extracted that explained 63.20% of the variance of the data. The cutting point for selecting variables on the extracted factors was 0.45 (Table 3).

**Table 3:** The total variance explained by the six-factor model

Factors	Elemental value			Sum of squares of factorized loads extracted		
	Total	Percentage of variance	Cumulative percentage	Total	Percentage of variance	Cumulative percentage
1	10.23	33.01	33.01	4.96	15.99	15.99
2	2.93	9.44	42.45	3.26	10.49	26.49
3	3.31	7.45	49.89	3.22	10.39	36.88
4	1.67	5.34	55.24	3.21	10.35	47.23
5	1.28	4.13	59.36	2.91	9.39	56.62
6	1.19	3.84	63.20	2.04	6.58	63.20

According to the results of factor analysis, 31 of the remaining main variables in the study (with a mean above 3) follow a six-factor model; subsequently, each of the variables was assigned in one factor, respectively, and the categories were named according to the content and the nature of the variables. Subsequently, the main areas of interest and their subset variables and relevant statistical information are presented separately. Thus, 15.901, 10.499, 10.399, 10.349, 9.389, and 6.584% of variance are explained by implementation, evaluation, content, structural, psychological, and managerial factors, respectively (Table 4).

**Table 4:** Statistics on extracted factors and related variables

Factors	Items	Mean	Factor Loading	Explained variation	Cronbach’s alpha
Implementation	Idealistic standards	4.23	0.502	15.901	0.898
	Tunnel vision	3.75	0.598		
	Unreal presentation of facilities	3.51	0.610		
	Rush in implementation	3.99	0.711		
	Lack of awareness personnel	3.55	0.654		
	Lack of cultural infrastructure	3.96	0.622		
	Shortage of staff	4.15	0.838		
	Lack of financial resources	3.81	0.766		
	Lack of commitment of doctors and managers	3.83	0.486		
Evaluation	Thought clash	3.56	0.665	10.499	0.815
	Subjective evaluation by inspectors	3.34	0.709		
	Bias in evaluation	2.95	0.780		
	Lack of skills of inspectors	3.18	0.677		
	The beneficiary of the evaluation device	2.74	0.507		
	Inappropriate behavior of inspectors	2.32	0.764		
Content	Disregard for results and attention to structures	3.65	0.736	10.390	0.793
	Inappropriate scoring scale	3.63	0.701		
	Disparities and balance of measures	3.72	0.702		
	Ignoring cross-department communications	3.22	0.511		
Structure	Emphasis on documentation	4.35	0.727	10.349	0.844
	The large number of standards	4.37	0.640		
	Ambiguity of measures	3.99	0.631		
	Paying less attention to patients	4.22	0.546		
	Disregard for the patients’ mental needs	3.49	0.571		
	Reducing the time of direct nursing care	3.98	0.617		
Psychological	Stress in the staff	4.30	0.737	9.389	0.806
	Physical and mental fatigue	4.30	0.831		
	Lack of time to implement standards	4.39	0.686		
Management	Integration of the evaluation and payment system	3.42	0.704	6.574	0.681
	Lack of proper financial feedback	3.86	0.509		
	Establishing unequal competition in hospitals	3.92	0.733		
	The sum of the variance explained			63.20	

## Discussion

The purpose of this study was to analyze the challenges of establishing the accreditation program in Iranian hospitals. The results indicate that there are numerous challenges in the successful implementation of the hospital accreditation program, which were categorized in six main factors including implementation, evaluation, content, structure, psychologic, and management. Lack of time to implement the standards, the large number of standards, emphasis on documentation, stress, physical and mental fatigue of the staff, paying less attention to patients, and shortage of staff were the main challenges of the hospital accreditation.

We found that lack of time was the most important challenge to implement the accreditation standards. As a result, accreditation in many hospitals has led to fake documentation and structural changes. To achieve positive outcome, hospitals need to refine structures and processes so that implementation of the standards requires to 2 or 3 years. In Ehlers et al.’s study, lack of time was seen as the most problematic barrier to the full benefits of Denmark accreditation programs [17]. Also, Yousefnejad et al. revealed that implementation of the standards and documentation took too much time of staff especially the nursing personnel [29].

Another challenge that influences the implementation of accreditation is the large number of standards. Although the number of standards has fallen from more than 8000 to 903 standards over three periods, it is still considered an important challenge. The large number of standards and criteria lead to more emphasis on documentation production rather than the process of implementing the standards. Therefore, it is necessary; MOH periodically reviews its standards using the literature review and consultation with experts, health care professionals, customers, academics, and policymakers. This challenge is parallel to those reported in other studies [29, 30]. The results of Nekoei-Moghadam et al.’s study showed that accreditation standards were more numerous [30].

Paying more attention to the documentation was found to be a major challenge in the implementation of accreditation. Standardizing and documenting processes, clarifying policies and roles, professional development, and using clinical guidelines for treating patients are some advantages [31, 32]. Hartley et al. (2002) confirmed the constructive role hospital written protocols and policies have on service provision [33]. However, in the current study, paying more attention to the documentation was identified as a main challenge. Iran’s accreditation depends mainly on documentation and degree orientation. Therefore, most hospitals produce fake documentation [30]. Different studies have shown that paying more attention to documentation is one of the barriers to effective implementation of accreditation [1, 24, 29, 30]. Bahadori et al. reported that focusing too much on documentation is one criticism leveled against the Iranian accreditation process [34]. In another study, nurses considered documentation boring, stressful, and an extra activity [35]. Intelligent computer systems, unifying and integrating policies and protocols by staff in academic centers, and decreases in extra bureaucracy can reduce documentation. Also, such bureaucratic processes can reflect weak human resource management (HRM), and solving this problem requires improvements in HRM processes, such as the establishing an appropriate and fair incentive system.

The implementation hospital accreditation places too much stress and physical and mental fatigue on hospital staff. Job stress and physical and mental fatigue was previously reported to accompany hospital accreditation [29, 36, 37]. Stress creates physical and mental problems for employees and negatively affects their quality of life [38, 39].

Implementation of accreditation standards requires sufficient staff. However, one of the challenges in implementing this program was staff shortages. Nurses are responsible for implementing the majority standards in Iranian hospitals. Insufficient nursing is a key challenge being faced by hospitals in Iran [38, 40]. Staff shortages were previously reported as potential barriers to accompany accreditation in hospitals in Iran [1, 25, 34] and other countries [23, 36]. Nekoei-Moghadam et al.’s study demonstrated that the Iranian accreditation system is not proportionate to the number of existing personnel, and this could cause the program to fail [30]. Pomey et al. revealed that, when a hospital is faced with staff shortages, accreditation is seen by staff as a time-consuming requirement that causes extra workload and stress [41]. Cerqueira in his review also stated that staff shortage leads hospitals to fail to achieve their accreditation goals [42]. Therefore, it is essential that hospital managers provide sufficient human resources to effective implementation of standards.

### Limitations

Our study has some limitations. First, our sample size was modest, but this limitation may be countered by the few number of factors that we examined, the relatively large number of items per factor, and the moderately high factor loadings. Thus, based on the simulation results by Mundfrom et al. [43], we believe our sample size was at least adequate. Second, our focus on hospital managers in Tehran may limit study generalizability to hospitals in other cities.

## Conclusions and recommendations

The present study showed that effective implementation of accreditation standards in Iranian hospital requires many changes in the field of structure, content, evaluation, psychological, management, and implementation. We suggest that managers and policymakers consider the challenges identified for effective implementation of accreditation. It is better to focus on challenges such as revising the standards, outcome centered standards, providing enough staff, localization of standards, and considering more time to enforce the standards. The results of this study may be considered as an evidence-based approach among managers and policymakers. However, similar studies are necessary elsewhere in the country.

## Data Availability

The datasets used and/or analyzed during the current study are available from the corresponding author on reasonable request.

## Appendix

**Table 5:** Descriptive statistics on the investigated challenges

Code	Variables	Mean	Standard deviation
1	The tastes of the evaluators and the unanimity of their opinions	3.56	0.944
2	The presence of insight and inspect mentality among evaluators and lack of counseling mentality	3.34	0.970
3	The existence of biases in accreditation assessments	2.95	0.981
4	Lack of adequate knowledge and assessment skills in some evaluators	3.18	0.986
5	The interest of the evaluator and its effect on the results (no outsourcing)	2.74	1.057
6	An inappropriate approach to assessors’ approach	2.32	0.972
7	Compact assessment period and insufficient number of days (evaluation within 1–2 days)	2.92	1.188
8	The reality of hospital evaluation scores in some cases	3.45	1.120
9	Too much emphasis on documenting and the possibility of obtaining a great deal only through the provision of documentation	4.35	0.878
10	A large number of measures and confusion of the staff	4.37	0.771
11	There is ambiguity in some measurements	3.99	0.962
12	Failure to pay attention to results and attention is paid to structures	3.65	0.956
13	Inappropriate scoring scale (e.g., 0–1–2) and lack of medium score	3.63	0.921
14	Imbalance and proportionality in measures	3.72	0..973
15	Disregarding communication between departments and interactions between units	3.22	0.983
16	Patient’s dissatisfaction with disregard for them because of the high amount of time required to be documented by nurses and physicians	4.22	0.959
17	Neglecting the spiritual needs of patients and need to treat them with dignity and respect	3.49	1.084
18	The lack of actual completion of forms and documentation by nurses and doctors due to their large volume	3.82	1.060
19	Reduce the duration of direct clinical care by nurses	3.98	0.995
20	Endangering occupational safety in the event of a low privilege in accreditation	3.14	1.185
21	Creating stress and anger in the staff from the pre-evaluation to the announcement of the results	4.30	0.828
22	Creating mental and physical fatigue in the staff	4.30	0.862
23	Lack of time to implement standards	4.39	0.801
24	The existence of idealistic and disproportionate standards with the current conditions of hospitals	4.23	0.880
25	Creating incentives to purchase unnecessary equipment through higher scores	3.41	1.131
26	Establishing a tunnel in the hospital and paying attention only to issues that affect the credibility of a higher score	3.74	1.010
27	Concealment and unrealistic display of hospital facilities	3.51	.1220
28	Forced to deceive the assessors	2.40	1.236
29	Mandatory implementation of hospital accreditation standards	3.92	1.039
30	The temptation and time compression in implementation and implementation of the program	3.99	0.946
31	The lack of staffing of the process and the reasons for the program	3.55	0.971
32	The lack of cultural infrastructures necessary for the implementation of accreditation (organizational culture, staff attitudes)	3.96	1.056
33	Shortage of human resources and increased staff turnover	4.15	1.111
34	Lack of financial resources for implementation of accreditation	3.81	1.188
35	Lack of back up resources such as books and sites to learn how to implement accreditation requirements	3.26	1.024
36	Lack of physical resources required for the implementation of standards, including equipment and physical space	3.65	1.098
37	Non-commitment and participation of some senior managers and physicians	3.83	1.077
38	Integration of the quality improvement and evaluation system with the payment system	3.42	1.048
39	Lack of proper feedback from surveys results and lack of financial incentives	3.83	1.031
40	The unequal competition between hospitals with different facilities and resources	3.92	1.104
	Total	3.64	0.558

## Abbreviations

I-CVI: Item-level content validity index
ISQua: International Society for Quality
KMO: Kaiser-Meyer-Olkin test

## References

[1] Reisi N, Raeissi P, Sokhanvar M, Kakemam E (2018). The impact of accreditation on nurses' perceptions of quality of care in Iran and its barriers and facilitators. Int J Health Plann Manag.

[2] Kakemam E, Dargahi H (2019). The health sector evolution plan and the technical efficiency of public hospitals in Iran. Iran J Public Health.

[3] Raeissi P, Sokhanvar M, Kakemam E (2018). Outsourcing in Iranian hospitals: findings from a qualitative study. Int J Health Plann Manag.

[4] Greenfield D, Braithwaite J (2009). Developing the evidence base for accreditation of healthcare organisations: a call for transparency and innovation. BMJ Qual Saf.

[5] Shaw CD, Braithwaite J, Moldovan M, Nicklin W, Grgic I, Fortune T (2013). Profiling health-care accreditation organizations: an international survey. Int J Qual Health Care.

[6] Braithwaite J, Shaw CD, Moldovan M, Greenfield D, Hinchcliff R, Mumford V (2012). Comparison of health service accreditation programs in low-and middle-income countries with those in higher income countries: a cross-sectional study. Int J Qual Health Care.

[7] Smits H, Supachutikul A, Mate KS (2014). Hospital accreditation: lessons from low-and middle-income countries. Glob Health.

[8] Al-Awa B, De Wever A, Melot C, Devreux I (2011). An overview of patient safety and accreditation: a literature review study. Res J Med Sci.

[9] Abdallah A, Haddadin BM, Al-Atiyat HM, Haddad LJ, Al-Sharif SL. Investigating the applicability of EFQM and KAIIAE in Jordanian healthcare organizations: a case study. Jordan J Mech Ind Eng 2013;7(1).

[10] Braithwaite J, Westbrook J, Pawsey M, Greenfield D, Naylor J, Iedema R (2006). A prospective, multi-method, multi-disciplinary, multi-level, collaborative, social-organisational design for researching health sector accreditation [LP0560737]. BMC Health Serv Res.

[11] Greenfield D, Hinchcliff R, Moldovan M, Mumford V, Pawsey M, Westbrook JI (2012). A multimethod research investigation of consumer involvement in Australian health service accreditation programmes: the ACCREDIT-SCI study protocol. BMJ Open.

[12] Jaafaripooyan E, Agrizzi D, Akbari-Haghighi F (2011). Healthcare accreditation systems: further perspectives on performance measures. Int J Qual Health Care.

[13] Fortune T, O’Connor E, Donaldson B. Guidance on designing healthcare external evaluation programmes including accreditation. Dublin, Ireland: International Society for Quality in Healthcare (ISQua). 2015. http://www.isqua.org/docs/default-source/archive%2D%2D-iap-reference-materials/isqua

[14] Saut AM, Berssaneti FT, Moreno MC (2017). Evaluating the impact of accreditation on Brazilian healthcare organizations: a quantitative study. Int J Qual Health Care.

[15] Lee MY (2014). Motivations to pursue accreditation in children's mental health care: a multiple case study. Nonprofit Management Leadership.

[16] Liu SH, Wu JN, Day JD, Muo CH, Sung FC, Kao CH (2015). Mortality and cost of radiation therapy for oesophageal cancer according to hospital accreditation level: a nationwide population-based study. Eur J Cancer Care.

[17] Ehlers LH, Jensen MB, Simonsen KB, Rasmussen GS, Braithwaite J (2017). Attitudes towards accreditation among hospital employees in Denmark: a cross-sectional survey. Int J Qual Health Care.

[18] Grepperud S (2015). Is the hospital decision to seek accreditation an effective one?. Int J Health Plann Manag.

[19] Hinchcliff R, Greenfield D, Westbrook JI, Pawsey M, Mumford V, Braithwaite J (2013). Stakeholder perspectives on implementing accreditation programs: a qualitative study of enabling factors. BMC Health Serv Res.

[20] Falstie-Jensen AM, Nørgaard M, Hollnagel E, Larsson H, Johnsen SP (2015). Is compliance with hospital accreditation associated with length of stay and acute readmission? A Danish nationwide population-based study. Int J Qual Health Care.

[21] Gorsuch RL (1997). Exploratory factor analysis: its role in item analysis. J Pers Assess.

[22] Gaskin CJ, Lambert SD, Bowe SJ, Orellana L (2017). Why sample selection matters in exploratory factor analysis: implications for the 12-item World Health Organization Disability Assessment Schedule 2.0. BMC Med Res Methodol.

[23] El-Jardali F, Hemadeh R, Jaafar M, Sagherian L, El-Skaff R, Mdeihly R (2014). The impact of accreditation of primary healthcare centers: successes, challenges and policy implications as perceived by healthcare providers and directors in Lebanon. BMC Health Serv Res.

[24] Gharibi F, Tabrizi JS. Challenges of the new Iranian accreditation system based on the requirements of the international society for quality in healthcare (ISQua). J Clin Res Governance. 2015;4(1):1-6.

[25] Saadati M, Yarifard K, Azami-Agdash S, Tabrizi JS (2015). Challenges and potential drivers of accreditation in the Iranian hospitals. Int J Hospital Res.

[26] Polit DF, Beck CT, Owen SV (2007). Is the CVI an acceptable indicator of content validity? Appraisal and recommendations. Res Nurs Health.

[27] DeVon HA, Block ME, Moyle-Wright P, Ernst DM, Hayden SJ, Lazzara DJ (2007). A psychometric toolbox for testing validity and reliability. J Nurs Scholarsh.

[28] DeVellis RF (2016). Scale development: theory and applications: sage publications.

[29] Yousefinezhadi T, Mosadeghrad AM, Mohammad A, Ramezani M, SARI AA (2017). An analysis of hospital accreditation policy in Iran. Iran J Public Health.

[30] Nekoei-Moghadam M, Amiresmaili M, Iranemansh M, Iranmanesh M (2018). Hospital accreditation in Iran: a qualitative case study of Kerman hospitals. Int J Health Plann Manag.

[31] Greenfield D, Braithwaite J (2008). Health sector accreditation research: a systematic review. Int J Qual Health Care.

[32] Touati N, Pomey M (2009). Accreditation at a crossroads: are we on the right track?. Health Policy.

[33] Hartley A, Griffiths R, Saunders K (2002). An evaluation of clinical governance in the public health departments of the West Midlands Region. J Epidemiol Community Health.

[34] Bahadori M, Ravangard R, Alimohammadzadeh K (2015). The accreditation of hospitals in Iran. Iran J Public Health.

[35] Sadeghi-Bazargani H, Tabrizi J, Saadati M, Hassanzadeh, Alizadeh G (2015). Nursing experiences of clinical governance implementation: a qualitative study. Clin Governance.

[36] Elkins G, Cook T, Dove J, Markova D, Marcus JD, Meyer T (2010). Perceived stress among nursing and administration staff related to accreditation. Clin Nurs Res.

[37] Manzo BF, Brito MJM, Corrêa AR (2012). Implications of hospital accreditation on the everyday lives of healthcare professionals. Rev esc enferm USP.

[38] Kakemam E, Raeissi P, Raoofi S, Soltani A, Sokhanvar M, Visentin DC (2019). Occupational stress and associated risk factors among nurses: a cross-sectional study. Contemp Nurse.

[39] Chegini Z, Asghari Jafarabadi M, Kakemam E (2019). Occupational stress, quality of working life and turnover intention amongst nurses. Crit Care Nurse.

[40] Zarea K, Negarandeh R, Dehghan-Nayeri N, Rezaei-Adaryani M (2009). Nursing staff shortages and job satisfaction in Iran: issues and challenges. Nurs Health Sci.

[41] Pomey M-P, Contandriopoulos A-P, François P, Bertrand D (2004). Accreditation: a tool for organizational change in hospitals?. Int J Health Care Qual Assur.

[42] Cerqueira M. A literature review on the benefits, challenges and trends in accreditation as a quality assurance system. Victoria, British Columbia: Ministry of Children and Family Development 2006. https://dspace.library.uvic.ca/bitstream/handle/1828/1497/cerqueira_marcos.pdf

[43] Mundfrom DJ, Shaw DG, Ke TL (2005). Minimum sample size recommendations for conducting factor analyses. Int J Test.

